# Investigation of urediospore morphology, histopathology and epidemiological components on wheat plants infected with UV‐B‐induced mutant strains of *Puccinia striiformis* f. sp. *tritici*

**DOI:** 10.1002/mbo3.870

**Published:** 2019-05-17

**Authors:** Yaqiong Zhao, Pei Cheng, Tingting Li, Jinxing Ma, Yuzhu Zhang, Haiguang Wang

**Affiliations:** ^1^ Department of Plant Pathology, College of Plant Protection China Agricultural University Beijing China

**Keywords:** epidemiological component, histopathology, pathogen morphology, *Puccinia striiformis* f. sp. *tritici*, UV‐B radiation, virulence variation

## Abstract

Planting resistant cultivars is the most economical and effective measure to control wheat stripe rust caused by *Puccinia striiformis* f. sp. *tritici* (*Pst*), but the cultivars often lose their resistance due to the emergence of new physiological races. The UV‐B‐irradiated urediospores of the *Pst* physiological race CYR32 in China were inoculated on wheat cultivar Guinong 22 for screening virulence‐mutant strains. CYR32 and mutant strains (CYR32‐5 and CYR32‐61) before and after UV‐B radiation were used to conduct urediospore morphological and histopathological observations and an investigation of epidemiological components. The results showed that UV‐B radiation affected the urediospore morphology of each strain. UV‐B radiation inhibited urediospore invasion and hyphal elongation, which mainly manifested as decreases in germination rate, quantities of hyphal branches, haustorial mother cells and haustoria and hyphal length. After wheat cultivar Mingxian 169 was inoculated with the UV‐B‐irradiated urediospores, the incubation period was prolonged, and the infection efficiency, lesion expansion rate, total sporulation quantity and area under the disease progress curve were reduced. The results demonstrated that CYR32‐5 and CYR32‐61 may have more tolerance to UV‐B radiation than CYR32. The results are significant for understanding mechanisms of *Pst* virulence variations and implementing sustainable management of wheat stripe rust.

## Graphical abstract

Two virulence mutant strains named as CYR32‐5 and CYR32‐61 were obtained from a dominant physiological race CYR32 of *Puccinia striiformis* f. sp. *tritici* (*Pst*) in China, verifying that UV‐B radiation can induce virulence variations of *Pst*. The three *Pst* strains (CYR32, CYR32‐5 and CYR32‐61) before and after UV‐B radiation were used to systematically investigate the changes in the aspects of spore morphology, histopathology and epidemiological components of wheat stripe rust. The results demonstrated that the virulence mutant strains CYR32‐5 and CYR32‐61 had more tolerance to UV‐B radiation than the original strain CYR32.

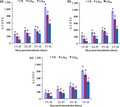

## 1 INTRODUCTION

Wheat stripe rust caused by *Puccinia striiformis* f. sp. *tritici* (*Pst*) is a worldwide airborne disease with important economic significance (Chen, Wellings, Chen, Kang, & Liu, 2014; Chen, 2005; Li & Zeng, 2002; Line, 2002; Wan, Chen, & He, 2007; Wang et al., 2014). The utilization of disease‐resistant cultivars is the most economical and effective measure to control wheat stripe rust with characteristics of rapid spread speed, widespread range and potential to cause severe damage (Li & Zeng, 2002). However, *Pst* can produce new pathotypes and physiological races via virulence variations, resulting in the breakdown of resistance to wheat stripe rust in resistant cultivars and periodic epidemics of the disease (Chen et al., 2014; Chen, 2007; Li & Zeng, 2002; Wan et al., 2004). *Pst* accomplishes a year‐round life cycle through airborne transmission of urediospores that are sensitive to temperature, humidity, light and other environmental conditions (Li & Zeng, 2002). Ultraviolet‐B (UV‐B, 280–315 nm) radiation is one of the influencing environmental factors that can induce virulence variation of *Pst* (Huang, Wang, Kang, & Zhao, 2005; Jing, Shang, & Li, 1993; Shang, Jing, & Li, 1994; Wang, Zhu, Huang, Wei, & Kang, 2009). The means of virulence variations of *Pst* mainly include sexual reproduction (Jin, Szabo, & Carson, 2010), heterokaryosis (Ma, Kang, & Li, 1993) and mutation (Li & Zeng, 2002). Many measures can be taken to induce *Pst* mutation (Hu, Wang, & Kang, 2014; Huang et al., 2005; Kang, Wang, Zhao, Tang, & Huang, 2015; Shang et al., 1994; Wang et al., 2009, 2013; Yao et al., 2006; Zhang, Wang, Wang, Jing, & Li, 2007). *Pst* can be induced to produce new pathogenic strains using artificial radiation‐induced mutation methods (Hu et al., 2014; Huang et al., 2005; Kang et al., 2015; Shang et al., 1994; Wang et al., 2009), among which UV‐B radiation is an important measure. Exploring the mechanisms of *Pst* virulence variation induced by UV‐B radiation can provide a scientific basis for understanding the “loss” of wheat resistance to stripe rust.

There are many reports on the effects of UV‐B radiation on plants (Quan et al., 2018; Rozema, van de Staaij, Björn, & Caldwell, 1997; Schreiner et al., 2012; Teramura & Sullivan, 1994; Wu et al., 2012) and pathogens (Braga, Rangel, Fernandes, Flint, & Roberts, 2015; Costa, Rangel, Morandi, & Bettiol, 2012; Li et al., 2018; Wargent & Jordan, 2013; Willocquet, Colombet, Rougier, Fargues, & Clerjeau, 1996). In particular, studies on the effects of UV‐B radiation on wheat stripe rust and *Pst* have also been reported (Cheng et al., 2014; Han, Wang, & Ma, 2011; Huang et al., 2005; Jing et al., 1993; Li, Han, & Ma, 2008; Shang et al., 1994; Wang, Qin, Cheng, Ma, & Wang, 2018; Wang et al., 2009). Han et al. (2011), Li et al. (2008) and Wang et al. (2018) investigated the effects of UV‐B radiation on the epidemiological components of wheat stripe rust on directly irradiated wheat seedlings. Jing et al. (1993) and Cheng et al. (2014) explored the changes of the epidemiological components of wheat stripe rust on wheat seedlings inoculated with UV‐B‐irradiated *Pst* urediospores.

Histopathological methods can be applied to investigate morphological changes of pathogens and plants at the cell and tissue levels during pathogen‐plant interactions, and the studies in this aspect can provide a basis for understanding pathogen infection processes and pathogen‐host interactions for disease control. In most related studies, histopathological observations were performed using light microscopy (Xavier, Alfenas, Matsuoka, & Hodges, 2001; Yin et al., 2015), electron microscopy (Kang & Buchenauer, 2000; Yin et al., 2015) and fluorescence microscopy (Han, Thieme, Gao, Kang, & Huang, 2013; Wesp‐Guterres, Martinelli, Graichen, & Chaves, 2013; Yin et al., 2015) to investigate the pathogenic processes of pathogens on host plants. As a kind of obligate parasite, *Pst* relies on living tissues of host plants to survive and complete life cycles. Therefore, it is particularly important to study *Pst*‐wheat interactions using histopathological methods. The methods have been applied to observe intercellular hyphal ultrastructure of *Pst* (Kang, Li, Shang, Chong, & Rohringer, 1993), *Pst* teliospore formation (Yao et al., 2012), infection structure of *Pst *in vitro (Wang, Zhang, Zhan, & Kang, 2010) and the ultrastructure of incompatible *Pst*‐wheat combinations (Kang, Wang, Huang, Wei, & Zhao, 2003) and to screen resistance genes based on wheat phenotype (Jagger, Newell, Berry, MacCormack, & Boyd, 2011), which has played significant roles in elucidating the *Pst*‐wheat interaction mechanism during infection process and the disease resistance mechanism.

To lay the foundations for exploring the virulence variation mechanism of *Pst* and to provide a scientific reference for wheat resistance breeding and control of wheat stripe rust, in this study, UV‐B radiation was used to treat *Pst* urediospores to screen virulence‐mutant strains of *Pst*. Then, the original strain and the obtained virulence‐mutant strains before and after UV‐B radiation were used to systematically investigate the changes in urediospore morphology, histopathology and epidemiological components of the disease.

## 2 MATERIALS AND METHODS

### 2.1 Materials and multiplication of *Pst* urediospores

CYR32, a dominant physiological race of *Pst* in China, was used. Multiplication of *Pst* urediospores was conducted on a winter wheat cultivar, Mingxian 169, that is highly susceptible to all known Chinese *Pst* physiological races. Chinese differential hosts including Trigo Eureka (T. E.), Fulhard, Lutesens 128, Mentana, Virgilio, Abbondanza, Early Premium, Funo, Danish 1, Jubilejina 2, Fengchan 3, Lovrin 13, Kangyin 655, Suwon 11, Zhong 4, Lovrin 10, Hybrid 46, *Triticum spelta album* and Guinong 22, were applied to determine the virulence characteristics of *Pst* strains. Using the method described by Cheng et al. (2014), wheat seedlings were incubated and *Pst* urediospores were multiplied in an artificial climate chamber (11–13°C, 60%–70% relative humidity, 12 hr light at an intensity of 10,000 lux per day).

UV‐B radiation of *Pst* urediospores was performed in a sealed light box made in‐house with three 36 W UV313 lamps (Beijing Lighting Research Institute, Beijing, China). The UV lamps were turned on for 30 min before irradiation and UV‐B intensity measurement using a UV‐B 297 radiation meter (Beijing Normal University Photoelectric Instrument Factory, Beijing, China). Different UV‐B radiation intensities were achieved by adjusting the distance between the *Pst* urediospores and the UV lamps.

### 2.2 Screening virulence‐mutant strains

To improve the probability of screening virulence‐mutant strains, according to the UV‐B radiation intensities acquired in the epidemic areas where virulence variation of *Pst* frequently occurs in China during the epidemic of wheat stripe rust (Cheng et al., 2014), a UV‐B radiation intensity of 250 μw/cm^2 ^was applied to irradiate the urediospores of CYR32. Using the method described by Cheng et al. (2014) and Zhao, Cheng, Li, and Wang (2018), the radiation time required for an approximately 90% relative lethal rate (i.e., the relative germination rate was approximately 10%) of the CYR32 urediospores under UV‐B radiation was selected as the optimal radiation time for screening virulence‐mutant strains. Repeated experiments showed that the relative lethal rate of the CYR32 urediospores was approximately 90% when the radiation time was 95 min. Therefore, the radiation time of 95 min was used as the optimal screening time in this study.

After 30 mg of urediospores of CYR32 were treated by UV‐B radiation for 95 min with an intensity of 250 μw/cm^2^, a suspension of the irradiated urediospores (0.6 mg/ml) was prepared with 0.2% Tween 80 solution and then artificially sprayed on 12 pots of Mingxian 169 seedlings, for which the first leaves were fully expanded. When the uredinia on the wheat leaves ruptured, the urediospores were collected and then inoculated artificially on the seedlings of Guinong 22 at the growth stage with the first fully expanded leaves. If stripe rust occurred on the seedlings of Guinong 22, the individual uredinia with infection types clearly different from those of the original strain, CYR32, were selected and then propagated throughout four successive generations using a single uredinium isolation method. A strain with stable infection type in the four successive generations was treated as a virulence‐mutant strain. The mutation rate was calculated using the following method.

In this study, the virulence mutation rate was calculated based on the number of germinated urediospores on the surface of wheat leaves. The seedlings of Guinong 22 were inoculated with the urediospores collected from Mingxian 169 infected with the UV‐B‐irradiated urediospores. At 24 hr post inoculation (hpi), leaf samples of Guinong 22 were collected. After the sampled leaves were treated using the living leaf transparency technique, the number of urediospores and the number of germinated urediospores on each leaf were observed directly using a light microscope at 100× (10 × 10) magnification (three replicates per treatment and at least five views for each replicate), and the average spore germination rate was calculated. A urediospore with a germ tube longer than the diameter of the urediospore was considered germinated. The virulence mutation rate of *Pst* was calculated with the following formula:

$$\text{MR}=\frac{\text{VMS}}{\text{NL}\times \text{NS}\times \text{SGR}}$$

where MR is the virulence mutation rate, VMS is the number of virulence‐mutant strains (i.e., the number of uredinia with stable infection types in the four successive generations on Guinong 22), NL is the number of leaves sampled from the inoculated wheat plants, NS is the average number of spores on an individual wheat leaf and SGR is the average spore germination rate.

The living leaf transparency was performed according to the following method. Each sampled wheat leaf was cut into segments with a length of approximately 2 cm and then put into a 40 ml fixation solution consisting of 95% ethanol and glacial acetic acid (v:v = 3:1). After 24 hr of fixation, the color of the leaf segments almost completely faded, and the segments were transferred into a solution that was prepared with 25 g of chloral hydrate and 10 ml of distilled water. After 24 hr, the leaf segments were completely transparent and then dyed for 20–30 min with a methyl blue solution consisting of 20 g phenol, 20 ml lactic acid, 40 ml glycerol, 0.05 g methyl blue and 20 ml distilled water.

### 2.3 Virulence assessment of *Pst* strains

Virulence determination of the original strain, CYR32, and the obtained virulence‐mutant strains was conducted using the 19 Chinese differential hosts described above. The seeds of 19 Chinese differential hosts and Mingxian 169 were sown in five 10 cm‐diameter pots with four cultivars per pot and 6–7 seeds per cultivar. When the first leaves were fully expanded, the seedlings of 19 Chinese differential hosts and Mingxian 169 were inoculated with a 0.5 mg/ml urediospore suspension prepared with 10 mg of fresh urediospores of each *Pst* strain and 20 ml of 0.2% Tween 80 solution. After 15 days, the symptoms of stripe rust appeared on the inoculated wheat leaves, and the infection type of each strain on each cultivar was assessed according to a 0–9 scale (Line & Qayoum, 1992), with 0–3 as the resistant type, 4–6 as the intermediate type and 7–9 as the susceptible type. Three replicate of virulence testing of each strain were performed.

### 2.4 Electron microscopic observation of the non‐UV‐B‐irradiated and UV‐B‐irradiated *Pst* urediospores

For the original strain, CYR32, and each obtained virulence‐mutant strain, morphological observations of the non‐UV‐B‐irradiated urediospores and the treated urediospores with UV‐B radiation under the dose for which the relative lethal rate of urediospores was 90% (i.e., UV‐B lethal dose 90%, LD_90_) were performed using a scanning electron microscope (SEM). Using the method described by Cheng et al. (2014) and Zhao et al. (2018), the radiation time required for a 90% relative lethal rate of urediospores of each strain under a UV‐B radiation intensity of 150 μw/cm^2 ^was determined. In this study, 3 mg of harvested fresh urediospores of each *Pst* strain was irradiated with a UV‐B radiation dose of LD_90_, and 3 mg of harvested fresh urediospores of the corresponding strain was treated as the control. For each *Pst* strain, some non‐UV‐B‐irradiated urediospores or UV‐B‐irradiated urediospores were evenly scattered on a piece of smooth‐surface weighing paper. A piece of adhesive copper foil tape with a length of 2 cm was used to stick urediospores. Then, the urediospores on the tape were sputter‐coated with gold and finally observed under the microscope.

### 2.5 Histopathological observation during the infection process of the *Pst* strains

Histopathological observations after inoculation with the non‐UV‐B‐irradiated and UV‐B‐irradiated urediospores of the original strain, CYR32, and the obtained virulence‐mutant strains were performed using the living leaf transparency technique described above. First, 40 mg of fresh urediospores of each strain was collected and divided into two groups (20 mg per group). Then 20 mg of urediospores was evenly scattered in a 10 cm‐diameter Petri dish and irradiated for 1 hr under a UV‐B radiation intensity of 250 μw/cm^2^. Subsequently, a suspension of the irradiated urediospores (1 mg/ml) was prepared and sprayed on three pots of Mingxian 169 seedlings with their first fully expanded leaves. Another three pots of the seedlings of Mingxian 169 with their first fully expanded leaves were inoculated with a suspension of 20 mg non‐UV‐B‐irradiated urediospores (1 mg/ml) and were treated as the control. At 6, 12, 24, 48, 72 and 96 hpi, five leaves with the same size were sampled from the seedlings inoculated with non‐UV‐B‐irradiated and UV‐B‐irradiated urediospores, respectively, and each leaf was cut into three segments with a length of 2 cm. After the transparency of the leaf segments was accomplished, histopathological observations were performed using an inverted fluorescence microscope. Meanwhile, the germination rates of urediospores were assessed, and the quantities of substomatal vesicles, haustorial mother cells, hyphal branches and haustoria were recorded.

### 2.6 Determination of the epidemiological components after inoculation with the non‐UV‐B‐irradiated and UV‐B‐irradiated urediospores of the *Pst* strains

To investigate the epidemiological components after inoculation with the non‐UV‐B‐irradiated and UV‐B‐irradiated urediospores of the original strain, CYR32, and the obtained virulence‐mutant strains, three treatments were set based on UV‐B radiation doses. For two of the three treatments, the urediospores were irradiated with LD_90_ and the radiation dose for which the relative lethal rate of urediospores was 50% (i.e., UV‐B lethal dose 50%, LD_50_) under a UV‐B radiation intensity of 150 μw/cm^2^, and these two treatments were recorded as the LD_90_ treatment and the LD_50_ treatment, respectively. For the other treatment, the urediospores were not irradiated with UV‐B, and this treatment was used as the control treatment (CK). The radiation time required for LD_90_ of each strain under a UV‐B radiation intensity of 150 μw/cm^2 ^was the same as that used for electron microscopic observation of the UV‐B‐irradiated urediospores of each strain described above. The radiation time required for LD_50_ of each strain under a UV‐B radiation intensity of 150 μw/cm^2 ^was determined using the method described by Cheng et al. (2014).

Three replicate were set for each treatment. For each replicate, a 0.1 mg/ml spore suspension prepared with 1 mg of the non‐UV‐B‐irradiated or UV‐B‐irradiated fresh urediospores of each *Pst* strain was sprayed on three pots of the seedlings of Mingxian 169, of which the first leaves were fully expanded. The epidemiological components, including incubation period, infection efficiency, lesion expansion rate, sporulation quantity and area under the disease progress curve (AUDPC), were investigated using the methods described previously (Cheng et al., 2014) with minor modifications. All significant difference analyses of the epidemiological components were performed with Duncan's multiple range tests at the level of 0.05 using the software IBM SPSS Statistics 21.0 (IBM Corp., Armonk, NY).

In this study, the incubation period referred to the days between the day on which the inoculation was conducted and the day on which the first uredinium ruptured. On the eighth day after inoculation, chlorotic spots appeared on the inoculated wheat leaves, and then daily observation of the wheat leaves was performed at the same time of day at which the inoculation was conducted until the day on which the first uredinium ruptured. Infection efficiency was calculated using the formula IE = IL/(Ns × LA), where IE is the infection efficiency, IL is the number of infection loci on each leaf, Ns is the number of urediospores per unit leaf area and LA is the leaf area. Before inoculation, the leaf area of Mingxian 169 was measured using a portable leaf area meter. When the inoculation was conducted, a Vaseline‐coated slide was placed beside the wheat leaves for microscopic examination of the number of urediospores per unit area at a magnification of 400× (10 × 40) in five views. When the disease symptoms appeared, the total number of infection loci on all leaves of a pot of wheat seedlings was investigated, and then the average on each leaf was treated as the value of IL. In this study, the lesion expansion rate was defined as the lesion expansion area per day. To determine the lesion expansion rate, a wheat leaf with only one lesion was selected from each replicate and labeled when the disease symptoms appeared on the inoculated wheat seedlings. The length and width of the lesion were measured, and the multiplication of these two values was performed to obtain the lesion area. The lesion was measured every other day until it stopped expanding. The lesion expansion rate was calculated using the formula LER = [(DLA*_i_*_ + 2_ − DLA*_i_*)/2]/DLA*_i_* × 100%, where LER is the lesion expansion rate, DLA is the disease lesion area and *i* is the days post inoculation (dpi). The overall lesion expansion rate across the entire lesion expansion period was calculated based on the lesion area measured for the first time and the area measured when the lesion stopped expanding. To assess sporulation quantity, a wheat leaf with only one lesion was chosen from each replicate and labeled when the inoculated wheat leaves of all treatments started to sporulate, and the urediospores produced on this leaf were collected using a test tube (10 cm in length and 1.2 cm in diameter). A urediospore suspension was prepared with the collected urediospores. Using a pipetting gun, 1 μL of suspension was collected for microscopic counting of the urediospores, and in this way, the urediospores in the 5 μL suspension were counted. The average of the urediospore amounts was treated as the number of urediospores per μL. Then, the sporulation quantity on the leaf was obtained according to the total volume of the urediospore suspension. The urediospore collections on this labeled leaf were performed every other day until no more spores were produced. The total sporulation quantity on the leaf was calculated by summing the sporulation quantities obtained in all urediospore collections across the entire sporulation period. To determine AUDPC, the disease incidence and disease severity of wheat stripe rust were surveyed every five days after the disease symptoms appeared according to the Rules for Monitoring and Forecast of the Wheat Stripe Rust (*Puccinia striiformis* West.) (National Standard of the People's Republic China, GB/T 15795–2011). The disease severity was classified as 1%, 5%, 10%, 20%, 40%, 60%, 80% or 100%. The value of AUDPC was calculated according to the method described by Cheng et al. (2014).

## 3 RESULTS

### 3.1 Screening and virulence testing results of mutant strains

**Figure 1:**
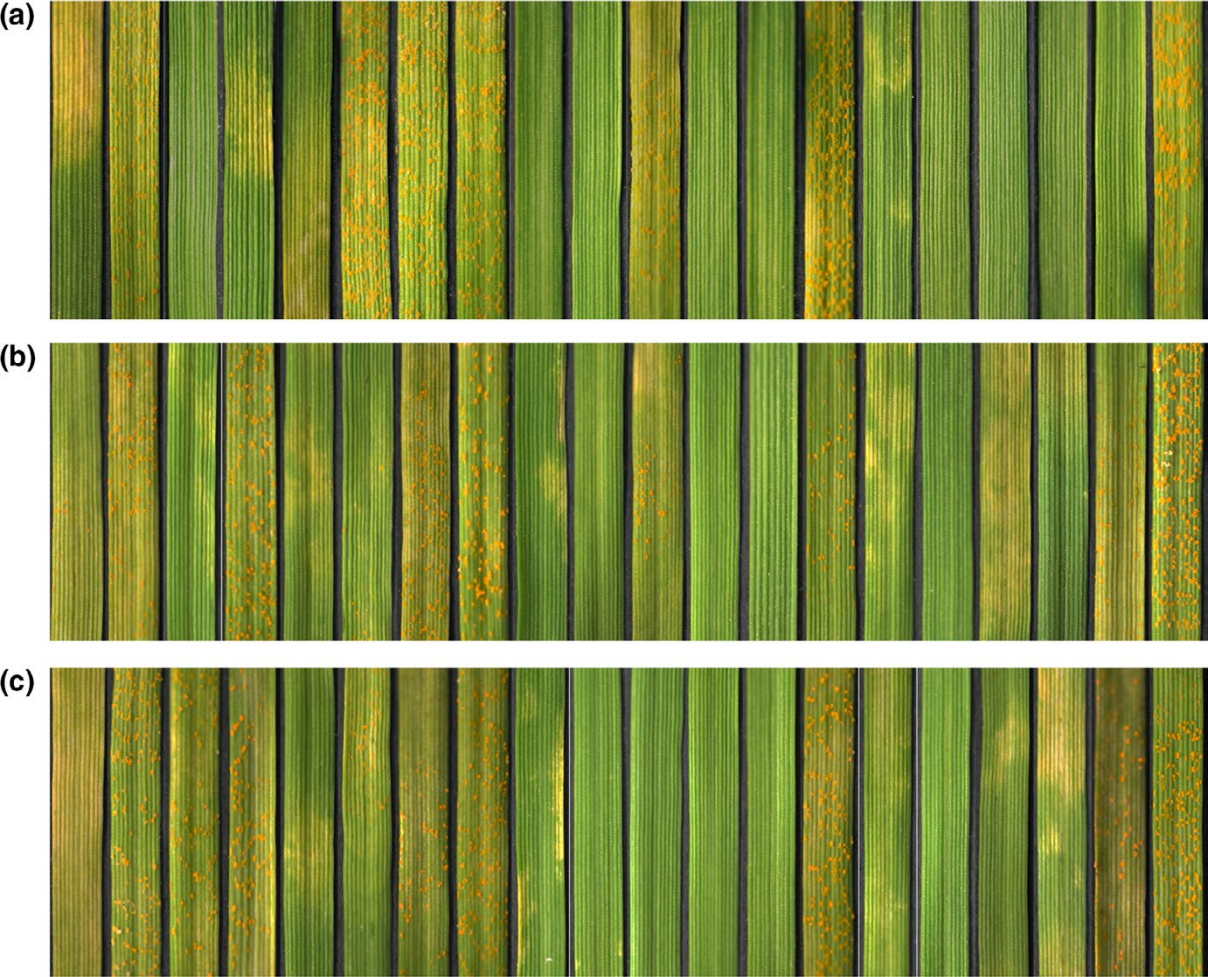
Infection types of CYR32 (a) and the two mutant strains CYR32‐5 (b) and CYR32‐61 (c) on Chinese differential hosts. In each row, the wheat cultivars from left to right were Trigo Eureka (T. E.), Fulhard, Lutesens 128, Mentana, Virgilio, Abbondanza, Early Premium, Funo, Danish 1, Jubilejina 2, Fengchan 3, Lovrin 13, Kangyin 655, Suwon 11, Zhong 4, Lovrin 10, Hybrid 46, *Triticum spelta album*, Guinong 22 and Mingxian 169

After screening strains on the seedlings of Guinong 22, two strains with stable infection types throughout the four successive generations were achieved and treated as the virulence‐mutant strains. The calculated virulence mutation rate was 5.37 × 10^−6^. These two mutant strains were named CYR32‐5 and CYR32‐61. The virulence determination results of mutant strains CYR32‐5 and CYR32‐61 on Chinese differential hosts are shown in Figure 1 and Table 1. The infection types of CYR32‐5 and CYR32‐61 on Guinong 22 were 6 and 7, respectively. Compared with the infection type of CYR32 on Guinong 22 (the infection type was 1), the virulence of CYR32‐5 and CYR32‐61 increased. Although as a naturally resistant cultivar to CYR32, Guinong 22 became susceptible to the two mutant strains. In addition, compared with CYR32, the virulence of CYR32‐5 increased on Mentana and decreased on Abbondanza, and the virulence of CYR32‐61 increased on Lutesens 128 and Mentana and decreased on Abbondanza and Fengchan 3. The results showed that the virulence of CYR32‐5 or CYR32‐61 on Guinong 22 was different from that of CYR32 on Guinong 22. The results also indicated that virulence variation of CYR32 could be induced by UV‐B radiation for 95 min under the intensity of 250 μw/cm^2^.

**Table 1:** Infection types of CYR32, CYR32‐5 and CYR32‐61 on Chinese differential hosts

Chinese differential host	Infection type		
	CYR32	CYR32‐5	CYR32‐61
Trigo Eureka (T. E.)	2	5	2
Fulhard	8	8	8
Lutesens 128	0	2	8
Mentana	2	8	9
Virgilio	2	2	2
Abbondanza	9	3	4
Early Premium	8	7	7
Funo	8	8	8
Danish 1	0	2	2
Jubilejina 2	0	1	0
Fengchan 3	7	5	0
Lovrin 13	0	0	0
Kangyin 655	0	0	0
Suwon 11	9	7	9
Zhong 4	2	2	2
Lovrin 10	1	0	0
Hybrid 46	1	2	1
*Triticum spelta album*	0	0	2
Guinong 22	1	6	7
Mingxian 169	9	9	9

### 3.2 Morphological observation results of the non‐UV‐B‐irradiated and UV‐B‐irradiated *Pst* urediospores using SEM

**Figure 2:**
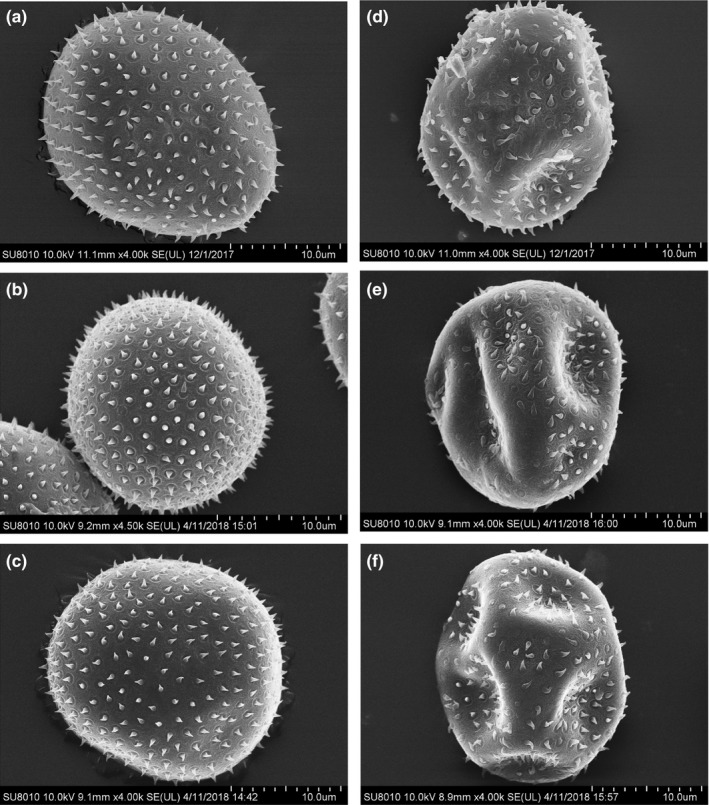
SEM morphological results of the non‐UV‐B‐irradiated urediospores of strains CYR32 (a), CYR32‐5 (b) and CYR32‐61 (c) and the urediospores of CYR32 (d), CYR32‐5 (e) and CYR32‐61 (f) irradiated with the dose for which the relative lethal rate of urediospores was 90%

Under a UV‐B radiation intensity of 150 μw/cm^2^, the radiation times required for LD_90_ of strains CYR32, CYR32‐5 and CYR32‐61 were 200, 300 and 230 min, respectively. For the three *Pst* strains, the SEM morphological observation results of the non‐UV‐B‐irradiated urediospores showed that the non‐UV‐B‐irradiated urediospores were spherical and the spines were intact and evenly distributed over the urediospores. However, irregular surface depressions appeared on the urediospores irradiated with UV‐B at a dose of LD_90_ under the intensity of 150 μw/cm^2^, the quantity of the spines over each urediospore became less, and some of them were broken (Figure 2). The results demonstrated that UV‐B radiation could impact the morphology of the *Pst* urediospores. The effects of UV‐B radiation on the morphology of the CYR32‐5 or CYR32‐61 urediospores were similar to those on the morphology of the CYR32 urediospores.

### 3.3 Histopathological observation results during the infection process of the *Pst* strains

**Figure 3:**
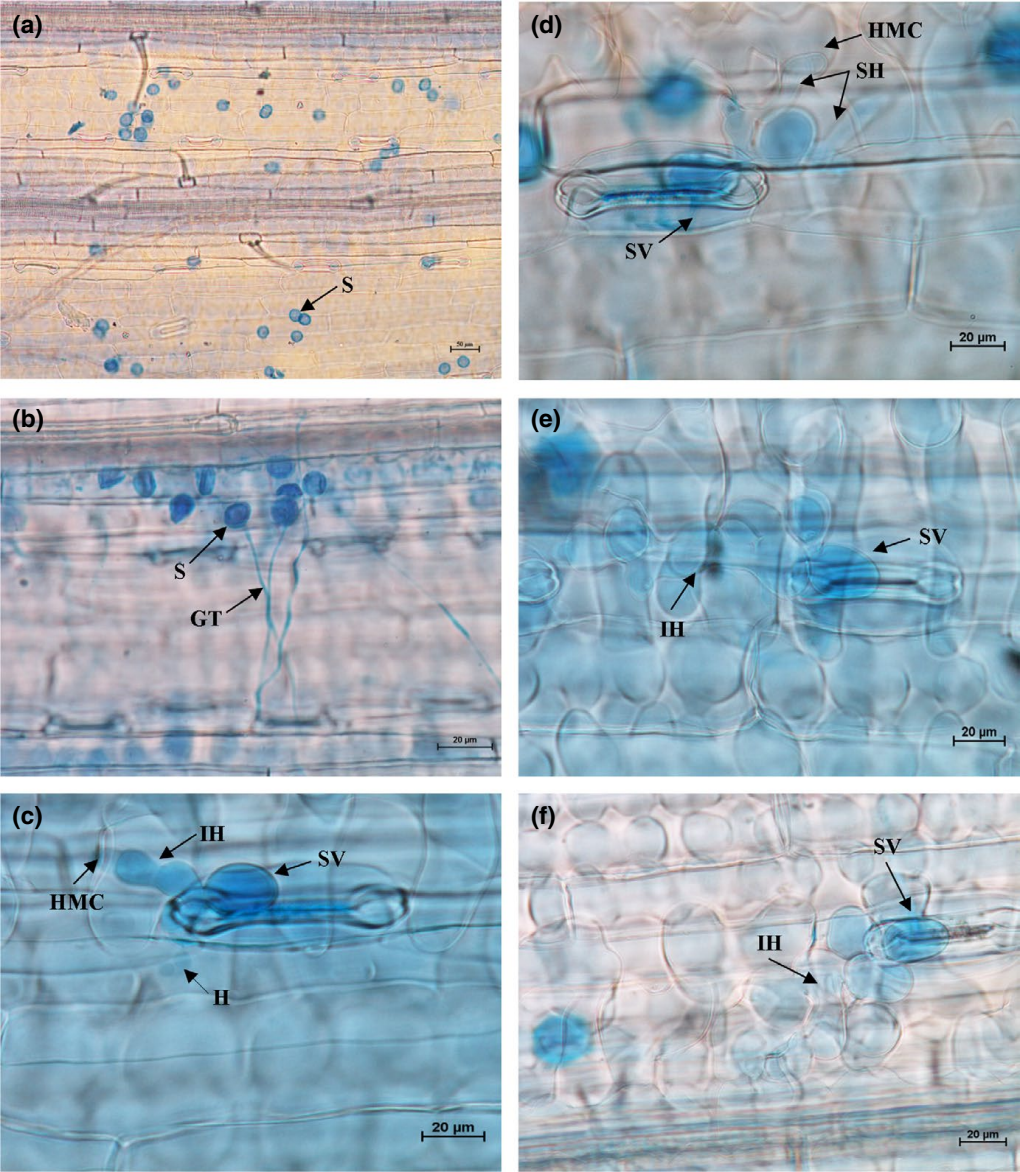
Histopathological results at different times after the seedlings of Mingxian 169 were inoculated with the non‐UV‐B‐irradiated urediospores of CYR32. (a) At 6 hpi, the urediospores began to germinate to produce germ tubes (GT); (b) At 12 hpi, most of the urediospores germinated, and only several substomatal vesicles (SV) were produced; (c) At 24 hpi, the germinated urediospores gradually produced SV, an SV produced two primary infection hyphae (IH), and haustorial mother cells (HMC) were formed at the hyphal tips and then invaginated into wheat mesophyll cells to form haustoria (H); (d) At 48 hpi, IH extended, and a few IH branched to produce secondary hyphae (SH); (e) At 72 hpi, both IH and SH continued to extend; (f) At 96 hpi, the hyphae extended, and a colony was formed. (a) Bar = 50 μm; (b)–(f) Bar = 20 μm

**Figure 4:**
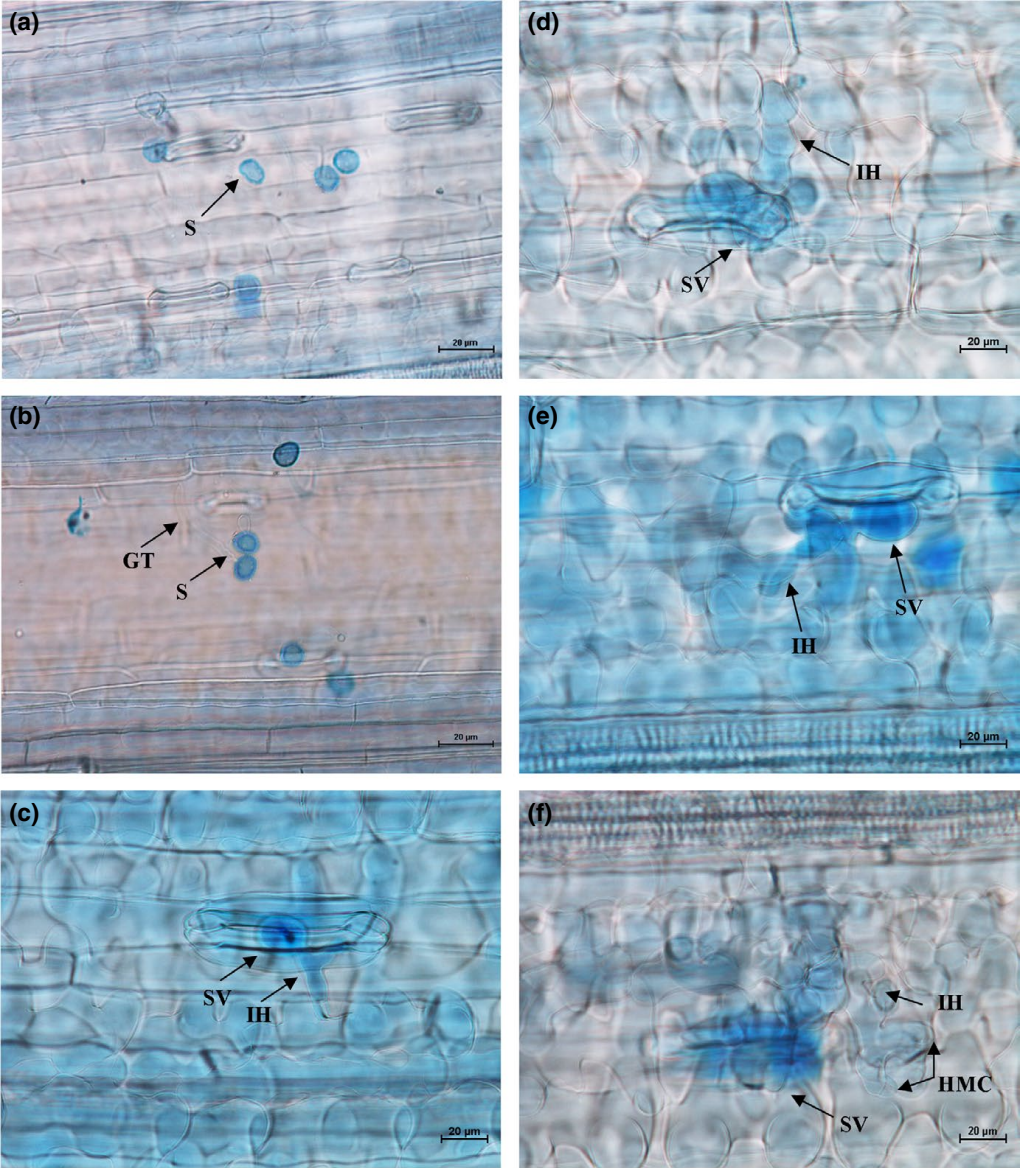
Histopathological results at different times after the seedlings of Mingxian 169 were inoculated with CYR32 urediospores irradiated under a UV‐B radiation intensity of 250 μw/cm^2^. (a) At 6 hpi, a few urediospores began to germinate to produce germ tubes (GT); (b) At 12 hpi, most of the urediospores germinated, and only several substomatal vesicles (SV) were produced; (c) At 24 hpi, the germinated urediospores gradually produced SV and two primary infection hyphae (IH) were produced from an SV; (d) and (e) At 48 and 72 hpi, IH remained on extension; (f) At 96 hpi, IH continued to extend and haustorial mother cells (HMC) were formed at the hyphal tips. (a–f) Bar = 20 μm

**Figure 5:**
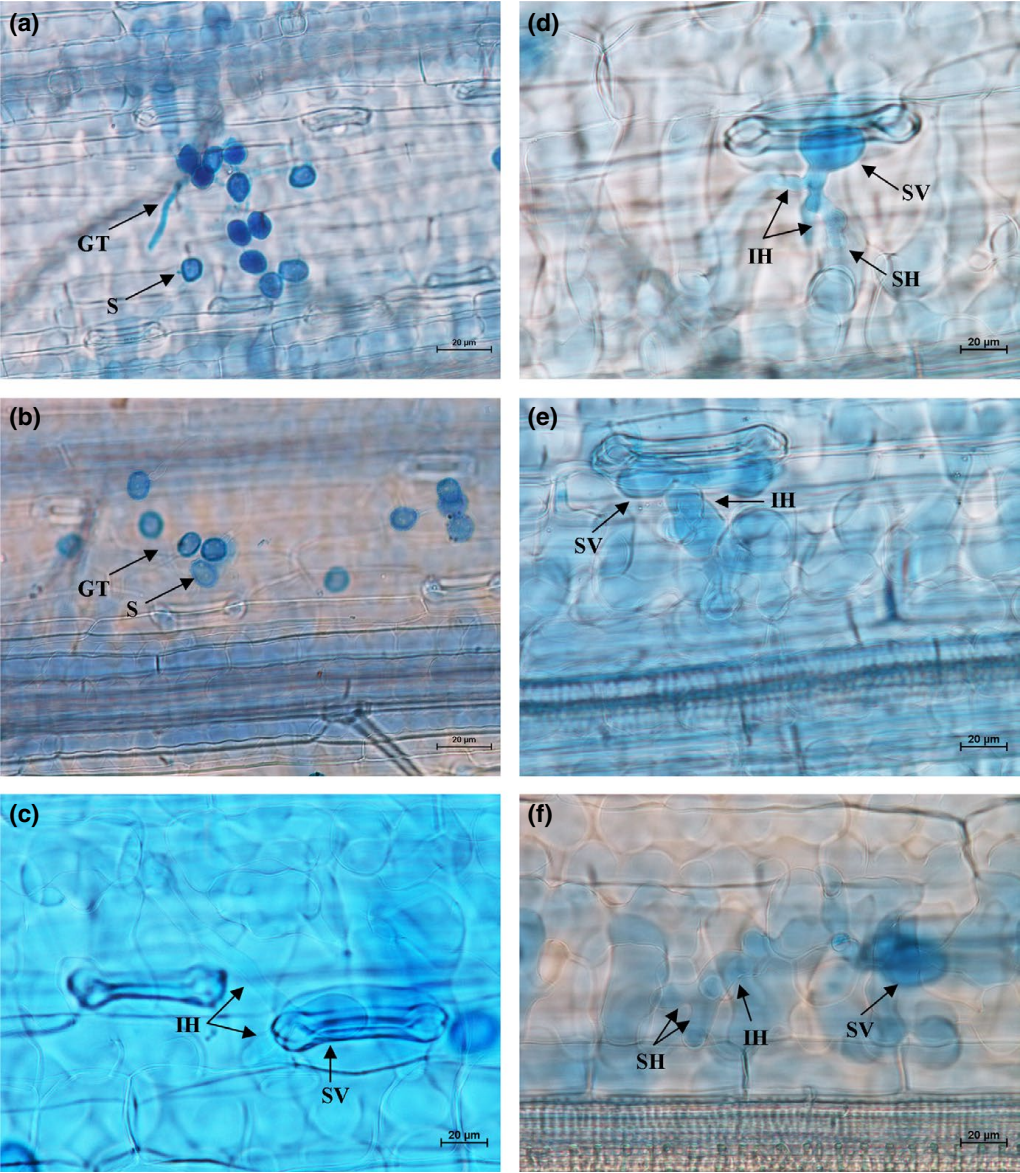
Histopathological results at different times after the seedlings of Mingxian 169 were inoculated with the non‐UV‐B‐irradiated urediospores of the mutant strain CYR32‐5. (a) At 6 hpi, the urediospores began to germinate to produce germ tubes (GT); (b) At 12 hpi, most of the urediospores germinated; (c) At 24 hpi, the germinated urediospores gradually produced substomatal vesicles (SV), an SV produced two primary infection hyphae (IH), and haustorial mother cells (HMC) were formed at the hyphal tips and then invaginated into wheat mesophyll cells to form haustoria (H); (d) At 48 hpi, IH extended and branched to produce secondary hyphae (SH); (e) At 72 hpi, IH and SH continued to extend. (f). At 96 hpi, IH and SH extended and formed a colony. (a–f) Bar = 20 μm

**Figure 6:**
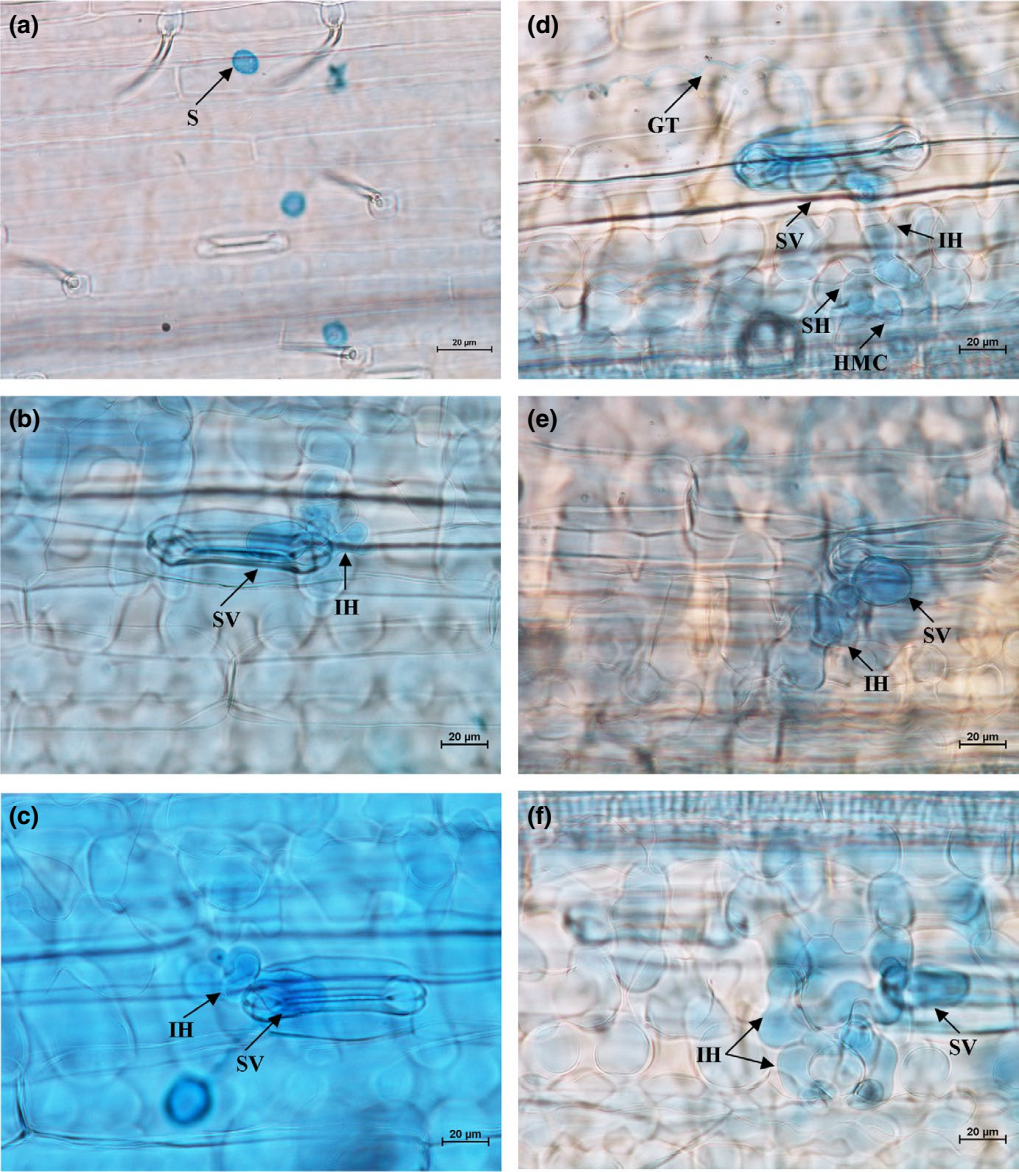
Histopathological results at different times after the seedlings of Mingxian 169 were inoculated with the urediospores of the mutant strain CYR32‐5 irradiated under a UV‐B radiation intensity of 250 μw/cm^2^. (a) At 6 hpi, a few urediospores were observed on the surface of wheat leaves; (b) At 12 hpi, some urediospores germinated, and only several substomatal vesicles (SV) were produced; (c) At 24 hpi, some germinated urediospores gradually produced SV, and an SV produced two primary infection hyphae (IH); (d) At 48 hpi, IH extended and branched to produce secondary hyphae (SH), and haustorial mother cells (HMC) were formed at the hyphal tips; (e) and (f) At 72 and 96 hpi, IH and SH continued to extend between host cells. (a–f) Bar = 20 μm

**Figure 7:**
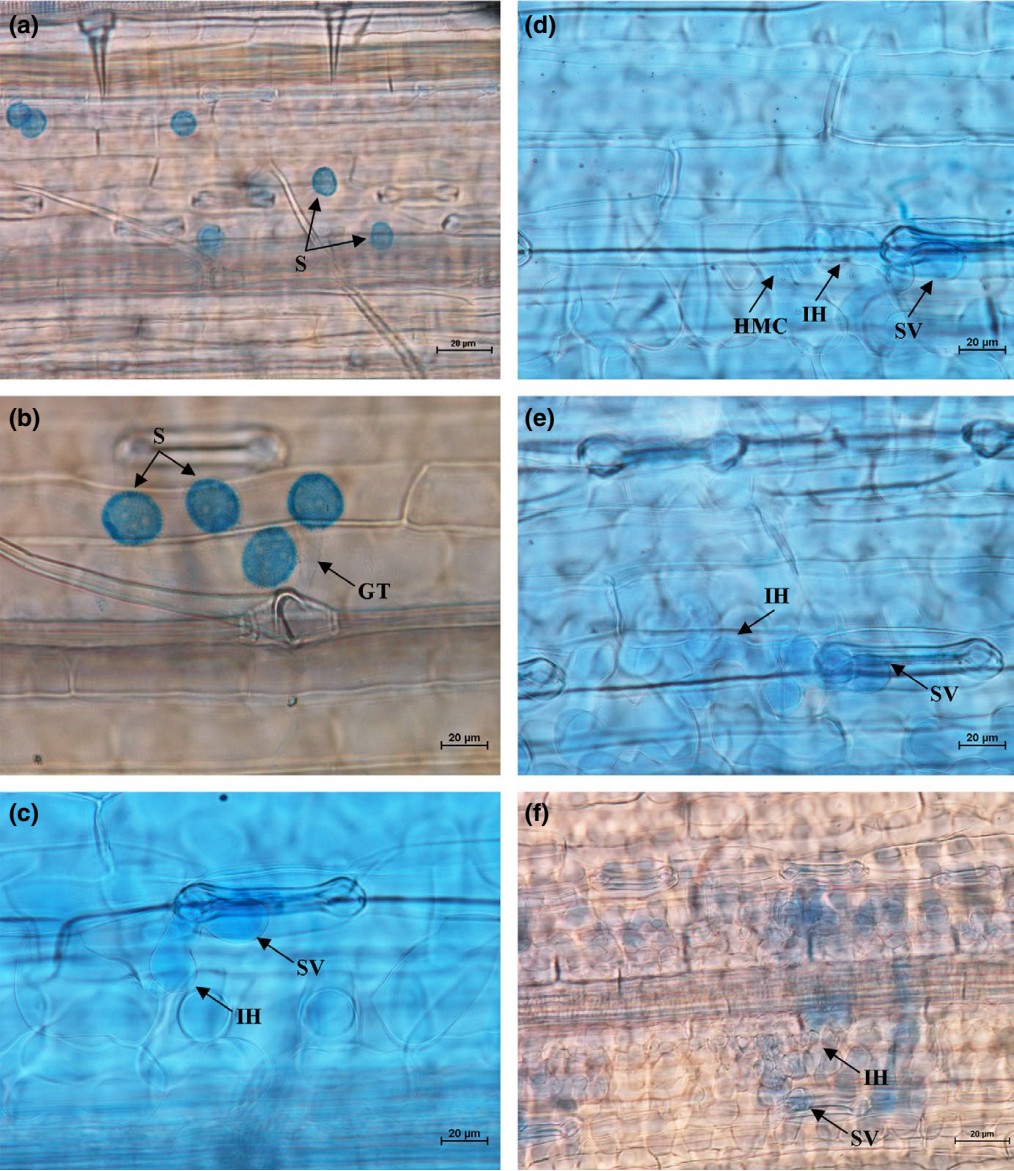
Histopathological results at different times after the seedlings of Mingxian 169 were inoculated with the non‐UV‐B‐irradiated urediospores of the mutant strain CYR32‐61. (a) At 6 hpi, the urediospores began to germinate to produce germ tubes (GT); (b) At 12 hpi, most of the urediospores germinated; (c) At 24 hpi, the germinated urediospores gradually produced substomatal vesicles (SV), and an SV produced one primary infection hypha (IH); (d) At 48 hpi, IH extended and haustorial mother cells (HMC) were formed at the hyphal tips; (e) At 72 hpi, IH extended and branched to produce secondary hyphae (SH); (f) At 96 hpi, both IH and SH continued to extend and formed a colony. (a–f) Bar = 20 μm

**Figure 8:**
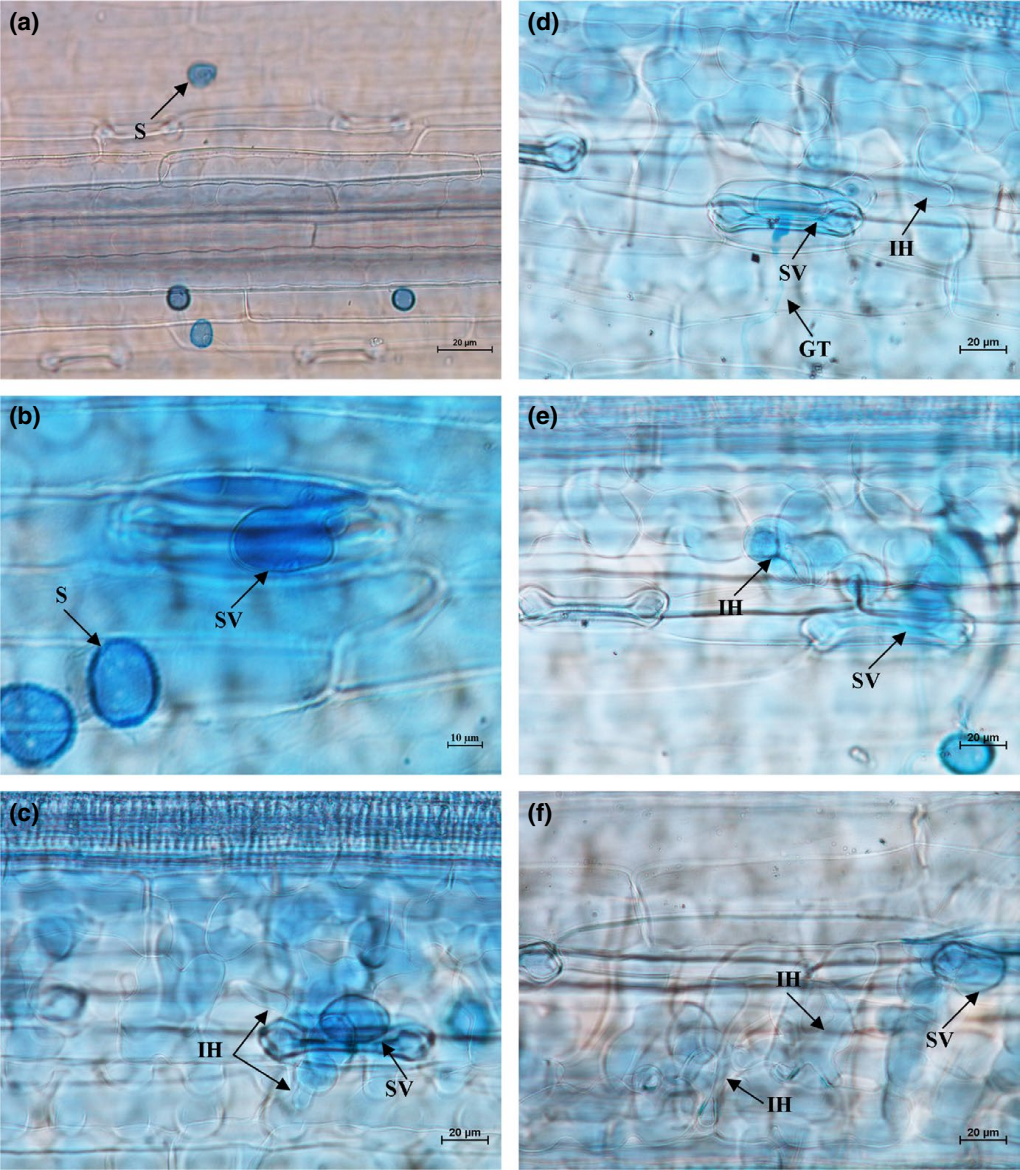
Histopathological results at different times after the seedlings of Mingxian 169 were inoculated with the urediospores of the mutant strain CYR32‐61 irradiated under a UV‐B radiation intensity of 250 μw/cm^2^. (a) At 6 hpi, a few urediospores were observed on the surface of wheat leaves, and only a small part of them germinated; (b) At 12 hpi, some urediospores germinated, and only several substomatal vesicles (SV) were produced; (c) At 24 hpi, several germinated urediospores produced SV, and an SV produced two primary infection hyphae (IH), one of which was produced from a hyphal swelling; (d) and (e) At 48 and 72 hpi, IH continued extending; (f) At 96 hpi, IH extended and branched to produce secondary hyphae (SH). (a–f) Bar = 20 μm

**Figure 9:**
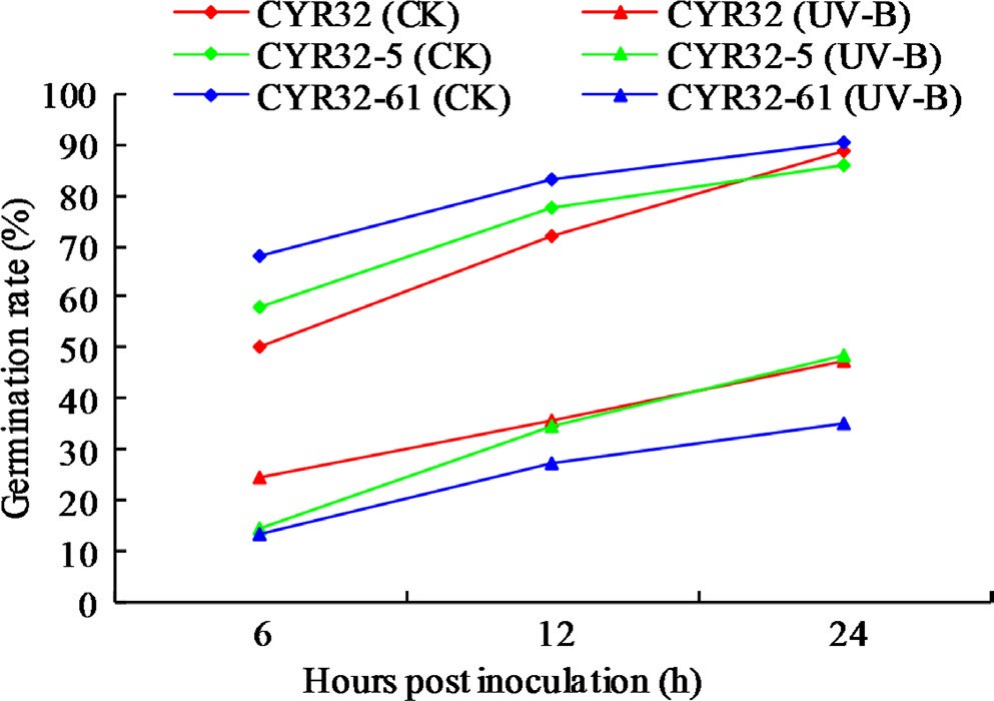
Germination rates of the non‐UV‐B‐irradiated and UV‐B‐irradiated urediospores of strains CYR32, CYR32‐5 and CYR32‐61 on the surface of Mingxian 169 leaves at different times after inoculation. CK between parentheses in the legend indicates that the urediospores were not treated by UV‐B radiation, and UV‐B between parentheses in the legend indicates that the urediospores were treated by UV‐B radiation with an intensity of 250 μw/cm^2^

**Figure 10:**
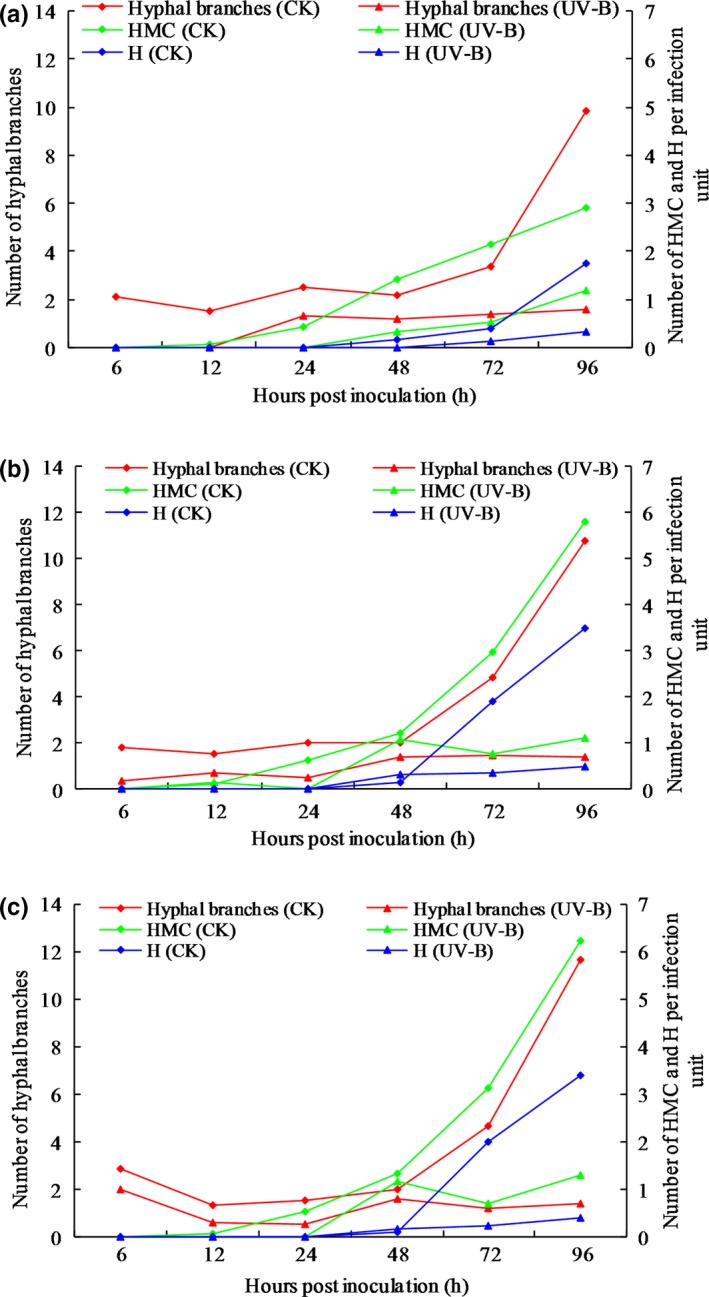
Changes in the numbers of hyphal branches, haustorial mother cells and haustoria per infection site in the leaves of Mingxian 169 at different times after inoculation with the non‐UV‐B‐irradiated and UV‐B‐irradiated urediospores of strains CYR32 (a), CYR32‐5 (b) and CYR32‐61 (c). CK between parentheses in the legend indicates the urediospores were not treated by UV‐B radiation, and UV‐B between parentheses in the legend indicates the urediospores were treated by UV‐B radiation with an intensity of 250 μw/cm^2^

Histopathological observation results at different times within 96 hr after the seedlings of wheat cultivar Mingxian 169 were inoculated with the non‐UV‐B‐irradiated and UV‐B‐irradiated urediospores of *Pst* strains CYR32, CYR32‐5 and CYR32‐61 showed that during the infection processes, there were differences among the three *Pst* strains in terms of the germination rates of urediospores and the quantities of substomatal vesicles, haustorial mother cells, haustoria and hyphal branches (Figures 3, 4, 5, 6, 7, 8). For each of the three *Pst* strain, the germination rate of the UV‐B‐irradiated urediospores was lower than that of the non‐UV‐B‐irradiated urediospores of the corresponding *Pst* strain (Figure 9). In addition, there was some delay in the germination of the UV‐B‐irradiated urediospores of each *Pst* strain in comparison to that of the non‐UV‐B‐irradiated urediospores of the corresponding *Pst* strain. At 24 hpi, the germination rates of the non‐UV‐B‐irradiated urediospores of CYR32, CYR32‐5 and CYR32‐61 were 89.0%, 85.9% and 90.3%, respectively, and those of the UV‐B‐irradiated urediospores of the corresponding strains were 47.6%, 48.3% and 35.0%, respectively. As shown in Figure 10, for each *Pst* strain, after UV‐B radiation under the intensity of 250 μw/cm^2^, both the germination rate of urediospores and the number of hyphal branches decreased, and the formations of haustorial mother cells and haustoria were inhibited. Histopathological observation results at 48, 72 and 96 hpi showed that with the development of *Pst* infection, the quantities of hyphal branches, haustorial mother cells and haustoria per infection site for each strain without UV‐B radiation increased rapidly and those for each strain after UV‐B radiation increased slowly. In contrast, the number of hyphal branches per infection site for each strain without UV‐B radiation increased with the greatest rate within 72–96 hpi. In particular, the quantities of the haustorial mother cells and the haustoria per infection site for the strains CYR32‐5 and CYR32‐61 without UV‐B radiation were greater than those of the non‐UV‐B‐irradiated CYR32 during the infection process, indicating that mutant strains CYR32‐5 and CYR32‐61 had stronger abilities in infection and expansion than the original strain, CYR32.

### 3.4 Investigation results of the epidemiological components after inoculation with the non‐UV‐B‐irradiated and UV‐B‐irradiated urediospores of the *Pst* strains

Under a UV‐B radiation intensity of 150 μw/cm^2^, the radiation times required for LD_90_ of the three *Pst* strains CYR32, CYR32‐5 and CYR32‐61 were the same as those used for electron microscopic observations of the UV‐B‐irradiated urediospores of the *Pst* strains described above, and those for LD_50_ of strains CYR32, CYR32‐5 and CYR32‐61 were 90, 180 and 120 min, respectively. The results demonstrated that the radiation times required for LD_90_ and LD_50_ of the two mutant strains CYR32‐5 and CYR32‐61 were longer than those required for the corresponding radiation doses of the original strain, CYR32. Among the three strains, the required UV‐B radiation time when the relative lethal rate of urediospores of CYR32‐5 reached 50% or 90% was the longest. For the control treatment, LD_50_ treatment and LD_90_ treatment, the investigation results of the epidemiological components, including incubation period, infection efficiency, lesion expansion rate, sporulation quantity and AUDPC, are shown below.

**Figure 11:**
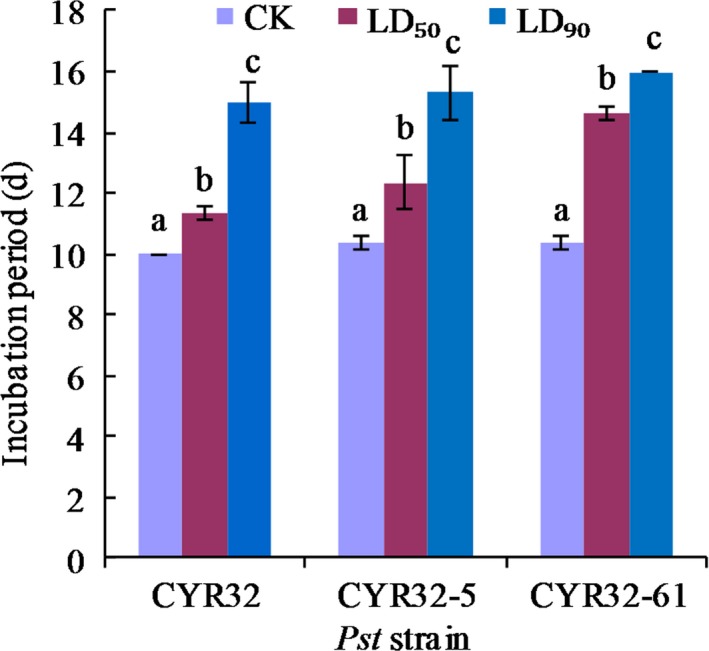
Effects of different doses of UV‐B radiation on the incubation periods of strains CYR32, CYR32‐5 and CYR32‐61. The different small letters indicate significant differences at the level of 0.05 (*p* < 0.05)

As shown in Figure 11, the incubation periods of CYR32, CYR32‐5 and CYR32‐61 under the LD_50_ treatment and the LD_90_ treatment were all prolonged and had significant differences (*p* < 0.05) from those of the corresponding strains under the control treatment. The incubation periods of CYR32, CYR32‐5 and CYR32‐61 under the control treatment were all 10 days. Under each treatment, no significant differences (*p* > 0.05) were found among the incubation periods of CYR32, CYR32‐5 and CYR32‐61. The results showed that the incubation period of each *Pst* strain under the LD_90_ treatment was significantly longer than that of the corresponding strain under the LD_50_ treatment.

**Figure 12:**
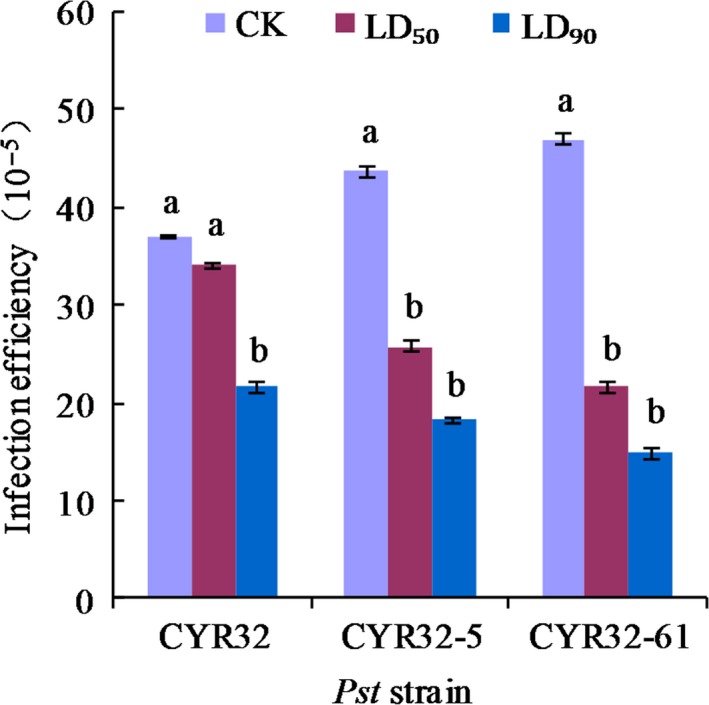
Effects of different doses of UV‐B radiation on the infection efficiencies of strains CYR32, CYR32‐5 and CYR32‐61. The different small letters indicate significant differences at the level of 0.05 (*p* < 0.05)

Investigation results of the infection efficiencies of the *Pst* strains are shown in Figure 12. The results showed that the infection efficiency of each strain decreased after UV‐B radiation in comparison to that of the corresponding strain under the control treatment. For CYR32, the infection efficiency under the LD_90_ treatments decreased significantly (*p* < 0.05) in comparison to that under the control treatment or the LD_50_ treatment, but there was no significant difference (*p* > 0.05) between the infection efficiencies under the control treatment and the LD_50_ treatment. For each of the mutant strains, the differences between the infection efficiencies of the control treatment and the LD_50_ treatment and between those of the control treatment and the LD_90_ treatment were significant (*p* < 0.05), but no significant difference (*p* > 0.05) was found between the infection efficiencies of the LD_50_ treatment and the LD_90_ treatment.

**Figure 13:**
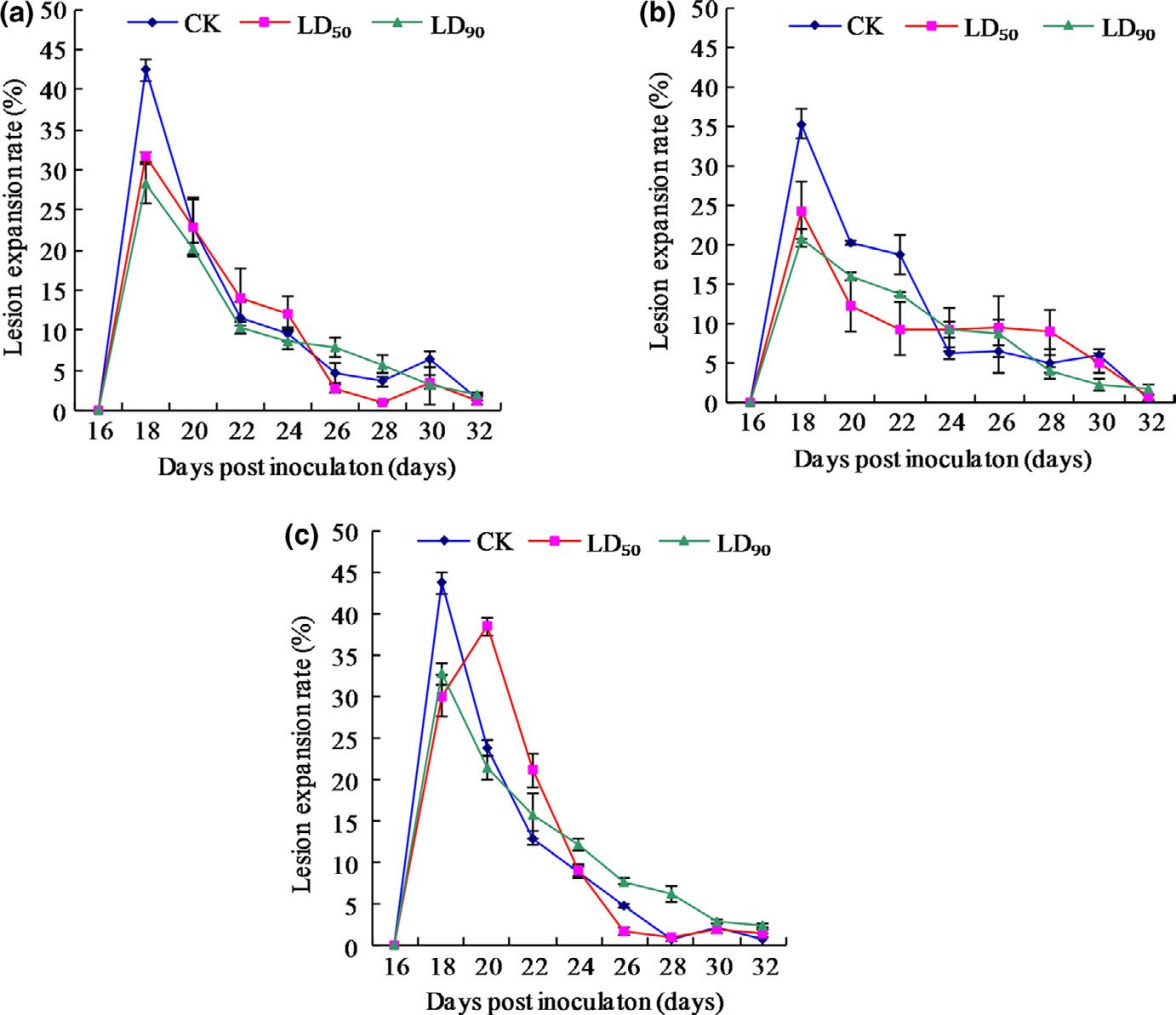
Changes of the lesion expansion rates on the leaves of Mingxian 169 inoculated with the urediospores of strains CYR32 (a), CYR32‐5 (b) and CYR32‐61 (c) treated with different doses of UV‐B radiation

The overall lesion expansion rates across the entire lesion expansion period for strains CYR32, CYR32‐5 and CYR32‐61 under the control, LD_50_ and LD_90_ treatments are shown in Table 2. For CYR32, the overall lesion expansion rates under both the LD_50_ treatment and the LD_90_ treatment significantly differed (*p* < 0.05) from those under the control treatment, but no significant difference (*p* > 0.05) was observed between the overall lesion expansion rates under the LD_50_ treatment and the LD_90_ treatment. For CYR32‐5 and CYR32‐61, the overall lesion expansion rates under both the LD_50_ treatment and the LD_90_ treatment did not significantly differ (*p* > 0.05) from those under the control treatment. However, with the increased UV‐B radiation dose, the overall lesion expansion rate for strain CYR32‐5 decreased and that for strain CYR32‐61 increased, indicating that strain CYR32‐61 may be more resistant to UV‐B radiation than strain CYR32‐5. The lesion expansion rates on the leaves of Mingxian 169 on different investigation dates after inoculation with the urediospores of strains CYR32, CYR32‐5 and CYR32‐61 under each treatment are shown in Figure 13. The calculated lesion expansion rate on the second investigation date was the maximum under each treatment for each strain except that the calculated lesion expansion rate under the LD_50_ treatment for strain CYR32‐61 reached the maximum on the third investigation date; after that, the lesion expansion rate demonstrated a descending trend until it reached 0.

**Table 2:** The overall lesion expansion rates across the entire lesion expansion period for strains CYR32, CYR32‐5 and CYR32‐61 treated with different doses of UV‐B radiation

*Pst* strain	Overall lesion expansion rates (%)		
	CK	LD_50_	LD_90_
CYR32	27.26 a	21.77 b	21.38 b
CYR32‐5	26.04 a	19.93 a	17.71 a
CYR32‐61	24.09 a	27.71 a	28.78 a

Note

The different small letters in the same row indicate significant differences at the level of 0.05 (*p* < 0.05).

**Figure 14:**
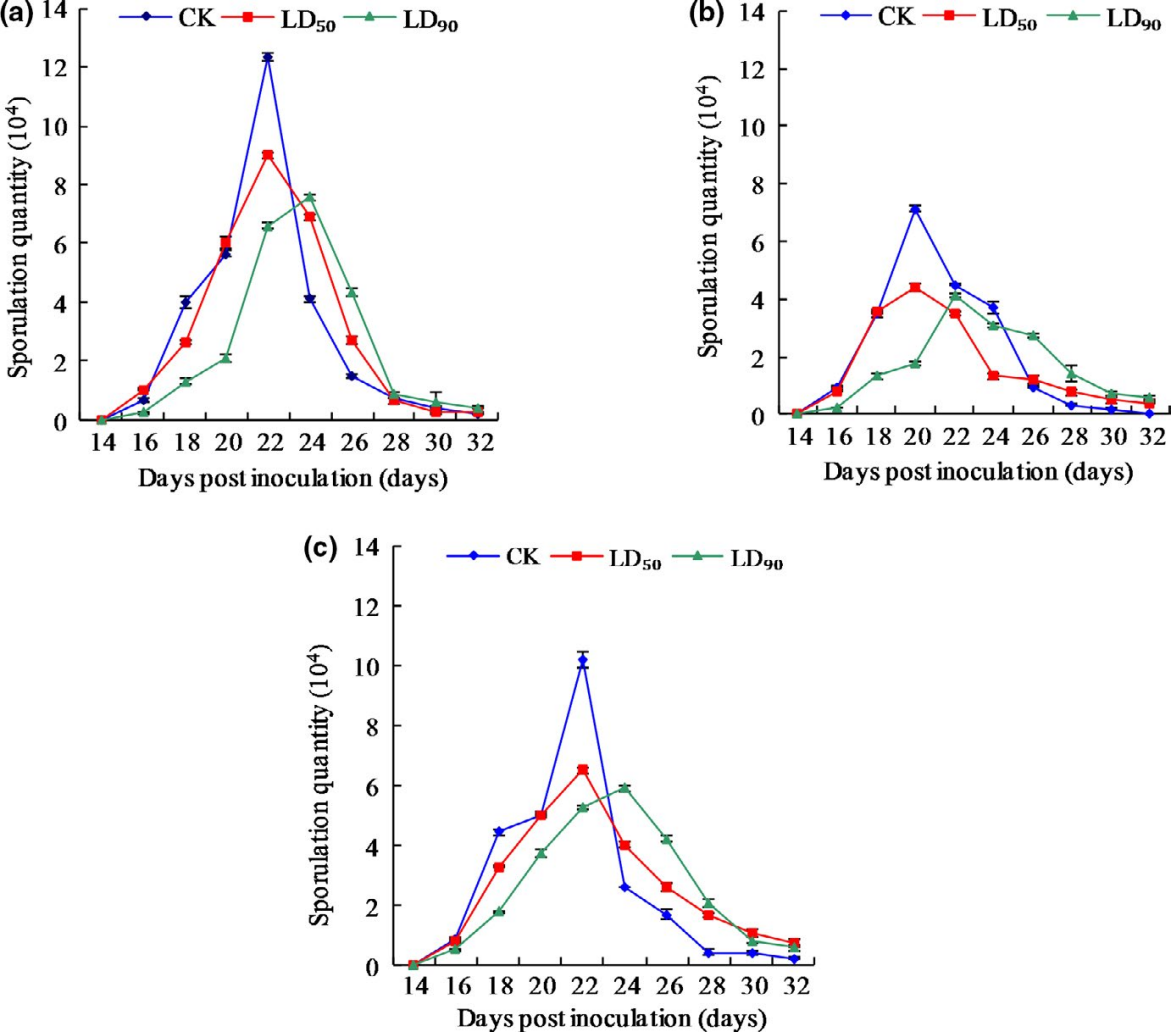
Dynamic changes of the sporulation quantities of strains CYR32 (a), CYR32‐5 (b) and CYR32‐61 (c) treated with different doses of UV‐B radiation

Results of the total sporulation quantities for strains CYR32, CYR32‐5 and CYR32‐61 are shown in Table 3. For CYR32, the total sporulation quantity under the LD_90_ treatment was significantly different (*p* < 0.05) from that under the control treatment or that under the LD_50_ treatment, but there was no significant difference (*p* > 0.05) between the total sporulation quantities under the control and LD_50_ treatments. For strain CYR32‐5, the total sporulation quantity under the LD_50_ treatment or the LD_90_ treatment was significantly different (*p* < 0.05) from that under the control treatment, but there was no significant difference (*p* > 0.05) between the total sporulation quantities under the LD_50_ treatment and the LD_90_ treatment. For strain CYR32‐61, no significant differences (*p* > 0.05) were found among the total sporulation quantities under the three treatments. Under each of the three treatments, the total sporulation quantity for strain CYR32‐5 was the minimum among the three strains. Different doses of UV‐B radiation had effects on the dynamic changes of the sporulation quantities of strains CYR32, CYR32‐5 and CYR32‐61 (Figure 14). For each strain, the time when the sporulation quantity under the LD_90_ treatment reached the maximum was delayed in comparison to that under the control treatment and the LD_50_ treatment. This result indicated that there may be a self‐repair process in each *Pst* strain after UV‐B radiation.

**Table 3:** The total sporulation quantities for strains CYR32, CYR32‐5 and CYR32‐61 treated with different doses of UV‐B radiation

*Pst* strain	Total sporulation quantity (×10^5^)		
	CK	LD_50_	LD_90_
CYR32	2.96 a	2.95 a	2.40 b
CYR32‐5	2.13 a	1.65 b	1.59 b
CYR32‐61	2.60 a	2.58 a	2.50 a

Note

The different small letters in the same row indicate significant differences at the level of 0.05 (*p* < 0.05).

**Figure 15:**
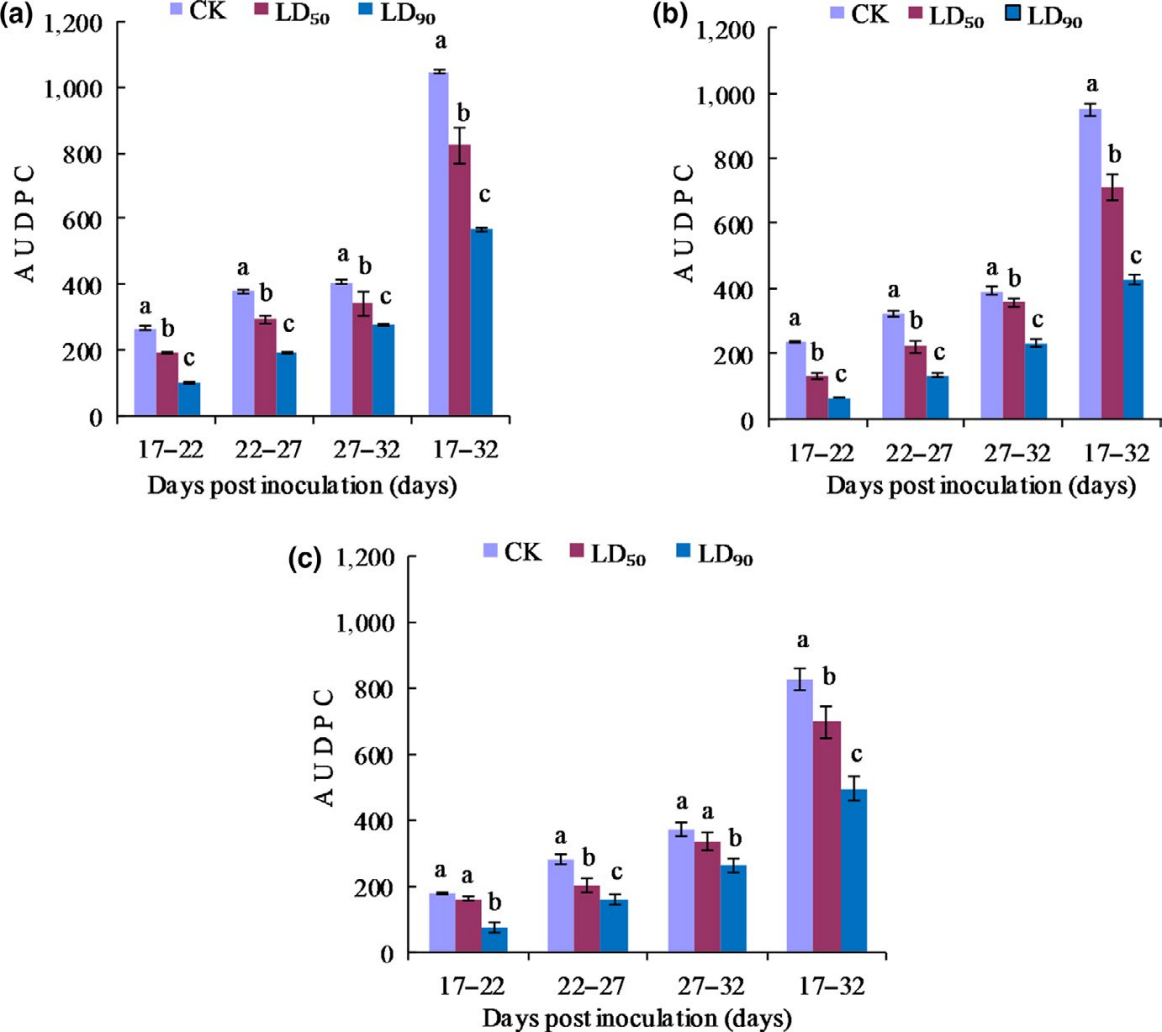
Effects of different doses of UV‐B radiation on the AUDPC of strains CYR32 (a), CYR32‐5 (b) and CYR32‐61 (c). The different small letters indicate significant differences at the level of 0.05 (*p* < 0.05)

For wheat stripe rust caused by strains CYR32, CYR32‐5 and CYR32‐61 under the control, LD_50_ and LD_90_ treatments, the results of the AUDPC values during the whole disease survey period (i.e., on 17–32 dpi) after the disease symptoms appeared are shown in Table 4. After each strain was irradiated by UV‐B, the AUDPC value decreased with increasing UV‐B radiation doses. For the each strain, there were significant differences (*p* < 0.05) among the AUDPC values under the three treatments. The AUDPC values of wheat stripe rust on Mingxian 169 inoculated with the non‐UV‐B‐irradiated and UV‐B‐irradiated urediospores of strains CYR32, CYR32‐5 and CYR32‐61 at different disease survey stages are shown in Figure 15. After the disease symptoms appeared, the AUDPC increased with the development of the disease. With the increase of UV‐B radiation dose, the AUDPC on 17–22, 22–27, 27–32 or 17–32 dpi for both CYR32 and CYR32‐5 significantly decreased (*p* < 0.05). For strain CYR32‐61, the AUDPC values on 17–22 or 27–32 dpi under the control treatment and the LD_50_ treatment had no significant difference (*p* > 0.05), but each of them significantly differed (*p* < 0.05) from the corresponding AUDPC under the LD_90_ treatment. There were significant differences (*p* < 0.05) among the AUDPC values on 22–27 or 17–32 dpi under the three treatments for the strain CYR32‐61. The results indicated that with increasing UV‐B radiation dose, CYR32‐5 may be more sensitive to UV‐B radiation than CYR32‐61.

**Table 4:** AUDPC values during the whole disease survey period (i.e., on 17–32 dpi) under the control, LD_50_ and LD_90_ treatments for strains CYR32, CYR32‐5 and CYR32‐61

*Pst* strain	AUDPC		
	CK	LD_50_	LD_90_
CYR32	1,048.17 a	824.24 b	565.96 c
CYR32‐5	948.75 a	708.29 b	428.31 c
CYR32‐61	826.01 a	696.06 b	493.42 c

Note

The different small letters in the same row indicate significant differences at the level of 0.05 (*p* < 0.05).

## 4 DISCUSSION

The aim of this study was to induce mutagenesis of CYR32 by UV‐B radiation, obtain virulence‐mutant strains and provide a basis for investigating the effects of UV‐B radiation on *Pst* and mechanisms of *Pst* virulence variations and for developing disease control strategies of wheat stripe rust. In this study, two virulence‐mutant strains, CYR32‐5 and CYR32‐61, were obtained from the screening cultivar Guinong 22. Both strains were able to infect Guinong 22, resulting in susceptible phenotypes. The virulence determination experiments showed that virulence variations of strains CYR32‐5 and CYR32‐61 also occurred on some other Chinese differential hosts. Successful acquisition of the virulence‐mutant strains laid a foundation for exploring the mechanisms of virulence variations of *Pst*.

Consistent with the previous SEM observations of the urediospores of *Puccinia* species (Littefiled, 2000; Traquair & Kokko, 1983), the non‐UV‐B‐irradiated urediospores of *Pst* strains CYR32, CYR32‐5 and CYR32‐61 used in this study were covered with spines. However, the UV‐B‐irradiated urediospores of the three strains CYR32, CYR32‐5 and CYR32‐61 irregularly invaginated, the quantity of the spines over each urediospore was reduced and some spines were broken. For the urediospores of strains CYR32, CYR32‐5 and CYR32‐61, the mechanism of the surface ultrastructural changes induced by UV‐B irradiation needs further research in the future.

In this study, to investigate the changes of infection processes after virulence variations of strain CYR32, histopathological observations were performed at different times after the seedlings of Mingxian 169 were inoculated with the non‐UV‐B‐irradiated and UV‐B‐irradiated urediospores of the three strains CYR32, CYR32‐5 and CYR32‐61. In comparison to the non‐UV‐B‐irradiated urediospores of each *Pst* strain, a delay phenomenon of urediospore germination was observed and the urediospore germination rate decreased after UV‐B radiation of the urediospores of the corresponding strain. The delay phenomenon of spore germination and the decrease of spore germination rate were also reported in other studies (Cheng et al., 2014; Costa, Gallego, & Tomaro, 2002; Costa et al., 2012). Spore germination of some plant pathogens can be inhibited and delayed by biotic factors or abiotic factors (Cheng et al., 2014; Costa et al., 2002, 2012). The inhibition of infection structure formation and the decrease in the quantity of each kind of infection structure for the UV‐B‐irradiated urediospores of each *Pst* strain may be due to the self‐repair of the UV‐B‐irradiated urediospores that were damaged by UV‐B radiation but not yet dead. These irradiated urediospores need time for self‐repair after inoculation, thus showing the delay phenomenon of the formation of infection structures (Cheng et al., 2014). The decrease in the quantity of each kind of infection structure for the UV‐B‐irradiated urediospores of each *Pst* strain may also be due to the decrease in the number of surviving urediospores resulting from direct killing of the *Pst* urediospores by UV‐B radiation (Cheng et al., 2014). However, the infection structures per infection site were investigated and used for analyses in this study, so the inconsistency of the numbers of surviving urediospores caused by UV‐B radiation could be ignored, and the latter reason could be excluded. Therefore, it is more likely that the formation inhibition and the quantity reduction of the infection structures were due to the former reason. Furthermore, compared with CYR32, the virulence‐mutant strains CYR32‐5 and CYR32‐61 produced more haustorial mother cells and haustoria per infection site, indicating that each virulence‐mutant strain had stronger infection ability than the original strain of *Pst*.

The investigation results of epidemiological components in this study showed that the incubation period of each of the three *Pst* strains was prolonged by UV‐B radiation, which was consistent with the report by Cheng et al. (2014). However, the difference in the incubation periods under the control treatment without UV‐B radiation between each of the two virulence‐mutant strains and the original strain, CYR32, was not significant (*p* > 0.05), indicating that virulence variations did not affect the incubation periods of the non‐UV‐B‐irradiated strains. The infection efficiencies of CYR32, CYR32‐5 and CYR32‐61 gradually decreased with increasing UV‐B radiation doses. In particular, for CYR32‐5 or CYR32‐61, there was no significant difference (*p* > 0.05) between the infection efficiencies under the LD_50_ and LD_90_ treatments. The results indicated that the sensitivity of the virulence‐mutant strains CYR32‐5 and CYR32‐61 to UV‐B radiation was lower than that of the original strain, CYR32. For CYR32‐61, the differences among the total sporulation quantities under the control, LD_50_ and LD_90_ treatments were not significant (*p* > 0.05). For CYR32, CYR32‐5 or CYR32‐61, the maximum daily sporulation quantity was reduced after UV‐B radiation, but the decline in sporulation quantity was delayed. After the sporulation quantity of each *Pst* strain reached its maximum, it decreased more slowly under the LD_50_ treatment or the LD_90_ treatment than under the control treatment. A similar trend was also demonstrated in the changes of the lesion expansion rates on the leaves of Mingxian 169 inoculated with the urediospores of the strains CYR32, CYR32‐5 and CYR32‐61 treated with different doses of UV‐B radiation. This phenomenon may be helpful to prolong the dispersal period of *Pst* urediospores under the natural environments, resulting in aggravation of wheat stripe rust. Therefore, it is of great significance to screen virulence‐mutant strains of *Pst* and explore mechanisms of virulence variations for investigating the epidemic trend of wheat stripe rust. AUDPC is an index that represents the cumulative development over time of plant disease. The greater the AUDPC value, the more sever the disease. The results in this study showed that UV‐B radiation reduced the AUDPC values of wheat stripe rust on Mingxian 169, indicating that UV‐B radiation could reduce the pathogenicity of the three *Pst* strains. The changes of the epidemiological components under the different UV‐B treatments for each of the three *Pst* strains indicated that the ranking of the three *Pst* strains may be CYR32‐61>CYR32‐5>CYR32 according to their tolerance to UV‐B radiation.

Beyond the investigations conducted in this study as described above, further studies should be carried out using the obtained virulence‐mutant strains of *Pst*. Studies on virulence‐mutant strains obtained using UV radiation at the molecular level were conducted by Huang et al. (2005) and Wang et al. (2009) using a random amplified polymorphic DNA technique, and the results showed that there were significant differences in DNA polymorphisms between mutant strains and wild‐type strains. Furthermore, the draft sequences of two *Pst* isolates have been reported (Cantu et al., 2011; Zheng et al., 2013). In further studies, in‐depth mining of the virulence‐related genes in virulence‐mutant strains of *Pst* and screening the mutation sites of the corresponding virulence‐related genes can be conducted for exploring the mechanisms of *Pst* virulence variations and breeding disease‐resistant wheat cultivars. In this study, two virulence‐mutant strains of *Pst* were obtained by UV‐B radiation, and some basis was provided for further studies.

## 5 CONCLUSIONS

In this study, two virulence‐mutant strains named CYR32‐5 and CYR32‐61 were obtained from the original strain, CYR32, via UV‐B radiation, verifying that UV‐B radiation can induce virulence variations of *Pst*. Morphological observation results obtained using SEM showed that UV‐B radiation could lead to morphological changes of the urediospores of the three *Pst* strains and induce destruction to the spines on the surfaces of the urediospores. Histopathological observation results during the infection process of the *Pst* strains showed that there were more haustorial mother cells and haustoria per infection site on Mingxian 169 inoculated with the virulence‐mutant strains CYR32‐5 and CYR32‐61 compared to that inoculated with the original strain, CYR32, which indicated that each of the virulence‐mutant strains had stronger infectivity than strain CYR32. The investigation results of epidemiological components showed that for strains CYR32, CYR32‐5 and CYR32‐61, after UV‐B radiation, the incubation period was prolonged, and the infection efficiency, lesion expansion rate, total sporulation quantity and AUDPC were reduced. The changes of the epidemiological components under the different UV‐B treatments for each *Pst* strain indicated that CYR32‐61 may have the strongest tolerance to UV‐B irradiation, followed by strain CYR32‐5, and the original strain, CYR32, may have the weakest tolerance among the three strains. The results in this study are of great significance for systematically understanding the effects of UV‐B radiation on urediospore morphology, the infection process and the epidemiological components of wheat stripe rust, revealing the mechanisms of virulence variations of *Pst* induced by UV‐B radiation and providing a basis for effective management of wheat stripe rust.

## CONFLICT OF INTERESTS

None declared.

## AUTHOR CONTRIBUTIONS

Conceptualization：Haiguang Wang; Data Curation：Yaqiong Zhao, Haiguang Wang; Formal Analysis： Yaqiong Zhao, Haiguang Wang; Funding Acquisition：Haiguang Wang; Investigation： Yaqiong Zhao, Pei Cheng, Tingting Li, Jinxing Ma, Yuzhu Zhang; Methodology： Yaqiong Zhao, Pei Cheng, Haiguang Wang; Project Administration： Haiguang Wang; Resources： Pei Cheng, Haiguang Wang; Supervision： Haiguang Wang; Visualization： Yaqiong Zhao, Haiguang Wang; Writing—Original Draft Preparation： Yaqiong Zhao, Haiguang Wang; Writing— Review & Editing： Yaqiong Zhao, Pei Cheng, Tingting Li, Jinxing Ma, Yuzhu Zhang, Haiguang Wang.

## ETHICS STATEMENT

None required.

## Data Availability

All relevant data are provided in full in the results section.
